# Crystal structure of the SH3 domain of human Lyn non-receptor tyrosine kinase

**DOI:** 10.1371/journal.pone.0215140

**Published:** 2019-04-10

**Authors:** Sandra Berndt, Vsevolod V. Gurevich, T. M. Iverson

**Affiliations:** 1 Department of Pharmacology, Vanderbilt University, Nashville, TN, United States of America; 2 Department of Biochemistry, Vanderbilt University, Nashville, TN, United States of America; 3 Vanderbilt Institute of Chemical Biology, Nashville, TN, United States of America; 4 Center for Structural Biology, Nashville, TN, United States of America; Yale University School of Medicine, UNITED STATES

## Abstract

Lyn kinase (**L**ck/**Y**es related **n**ovel protein tyrosine kinase) belongs to the family of Src-related non-receptor tyrosine kinases. Consistent with physiological roles in cell growth and proliferation, aberrant function of Lyn is associated with various forms of cancer, including leukemia, breast cancer and melanoma. Here, we determine a 1.3 Å resolution crystal structure of the polyproline-binding SH3 regulatory domain of human Lyn kinase, which adopts a five-stranded β-barrel fold. Mapping of cancer-associated point mutations onto this structure reveals that these amino acid substitutions are distributed throughout the SH3 domain and may affect Lyn kinase function distinctly.

## Introduction

Lyn kinase (**L**ck/**Y**es related **n**ovel protein tyrosine kinase) is a Src-family kinase organized into four domains. These include an N-terminal unique domain (also known as SH4 domain), two adapter domains (SH3 and SH2) and C-terminal kinase domain (also known as SH1 domain). Src-family kinase activity is conformationally regulated via distinct interactions between these domains. In the inactive state, the SH3 domain binds to the linker between the SH2 domain and the kinase domain, keeping the kinase in a closed (inactive) conformation [1]. In the active state the SH2 and SH3 domains interact with effectors proteins [2]. This releases the kinase domain and in the resulting open (active) conformation the kinase domain can phosphorylate its substrates [3, 4].

Active Src-family kinases phosphorylate both cytosolic and membrane-anchored proteins. Lyn substrates include, but are not limited to, β-catenin, N-myristoyl transferase 1, and transcription factor Stat3 [5, 6]. The total number of Lyn substrates is not known [7–10] but the physiological impact of substrate phosphorylation by Lyn kinase is cell growth and proliferation [11–13]. SH2 and SH3 adapter domains regulate the interactions of Lyn kinase with substrates and may help confer substrate specificity. Of these, the SH3 domain binds to polyproline sequence motifs (PxxP) in a way that can be recapitulated by peptides [14]. This suggests that the SH3 domain can induce a binding-competent backbone conformation within the region of sequence containing the PxxP motif [15, 16].

Lyn is overexpressed in the hematopoietic cells of patients with acute myeloid leukemia [17], and may be a major drug target for this leukemia type [13]. Lyn overexpression is also observed in colorectal, breast, renal and ovarian cancer [18–21]. Overexpression of Lyn kinase in lung cell carcinoma correlates with poor prognosis [22]. Mutations in Lyn kinase have been found in at least 17 cancer types, including breast, prostate, and liver cancer [23–25]. Due to the role of Lyn kinase in cancer, five Lyn inhibitors (Bosutinib, Ponatinib, Nintedanib, Dasatinib and Bafetinib) are used as therapeutics [26–32], with additional inhibitors, such as Saracatinib, currently in clinical trials [33]. These inhibitors target the active site within the kinase domain [34].

While there is utility in this approach, an additional therapeutic strategy could be the regulation of Lyn kinase activity via the SH3 domain [35]. This strategy requires clear understanding how the structure of the SH3 domain affects kinase activity. NMR structures of the Lyn SH3 domain in the presence and absence of a herpesvirus-derived polyproline-containing peptide previously identified how the Lyn SH3 domain interacts with a high-affinity ligand [36]. Here, we determined the structure of the Lyn SH3 domain to 1.3 Å resolution using X-ray crystallography, which allowed up to propose how cancer-associated point mutations affect this domain.

## Materials and methods

### 2.1 Expression and purification of the human Lyn SH3 domain

*E*. *coli* BL21 (*DE3*) cells were transformed with pDONR223-Lyn (Addgene plasmid # 23905) containing the coding sequence of SH3 domain of Lyn kinase in pET24(+) vector (Novagen). Cells were grown at 37°C in 1 L LB medium containing 100 mg/L ampicillin. At an OD_600_ of 0.8, expression was induced with 1 mM IPTG and the temperature was lowered from 37°C to 30°C. After 5 h, cells were harvested at 9,180 × *g*, and the pellet was resuspended in 50 mM Tris-HCl, pH 7.5, 0.5 mM EDTA and 50 mM NaCl (25 ml per 1 L of culture). Protease inhibitor cocktail (Sigma, 600 μl per 1 L cell culture), 2 mM MgCl_2_ and DNase 1 (20 U per 1 L of cell culture), 1 mM TCEP, and 100 mg/L lysozyme were added to the suspension. The suspension was sonicated (for 5 sec on/off cycle, 10 min, using a 70% power) and the lysate was centrifuged for 1h at 4°C at 38,360 × *g*. The supernatant was passed through a 0.45 μm filter, and loaded on a 5 ml His-Trap HP column equilibrated in wash buffer (50 mM Tris-HCl, pH 7.5, 150 mM NaCl, 10% glycerol and 15 mM imidazole). The protein was eluted in wash buffer containing 250 mM imidazole. Protein was further purified by size exclusion chromatography on a Superdex 200 10/300 GL column equilibrated with 20 mM Tris-HCl, pH 7.5, 5% glycerol, 150 mM NaCl, and 1 mM TCEP.

### 2.2 Crystallization and data collection

Crystals of purified Lyn SH3 domain grew by the hanging drop vapor diffusion method using 1.5 μl of Lyn SH3 domain (10 mg/ml in 20 mM Tris pH 7.5) equilibrated with 1.5 μl of reservoir solution (0.1 M Na citrate pH 3.5, and 3.2 M NaCl) and appeared within 24 h at 22°C. Crystals were cryo-protected using 0.09 M Na citrate pH 3.5, 2.88 M NaCl and 10% glycerol, and were flash cooled by plunging in liquid nitrogen. Data were collected at the Stanford Synchrotron Radiation Lightsource (SSRL) Experimental Station 9–2 using a Pilatus 6M detector (**Table 1**).

**Table 1:** Data collection and refinement statistics for the Lyn SH3 domain. Values in parentheses correspond to the highest resolution shell. For data collection, this corresponds to 1.22–1.20 Å resolution. For refinement, this corresponds to 1.26–1.20 Å resolution. Data are >95% complete at 1.45 Å resolution.

Data collection statistics	
SBGrid Entry	640
Resolution	1.20 Å
Space group	P6_1_
Unit-cell dimensions	a = 46.7 Å, b = 46.7 Å, c = 55.7 Å
R_sym_	0.038 (0.512)
R_pim_	0.012 (0.171)
I/σ	53.6 (4.00)
Completeness (%)	87.1% (81.8%)
Redundancy	10.5 (9.7)
CC_1/2_	0.994 (0.923)
**Refinement Statistics**	
PDB entry	6NMW
R_cryst_	0.172 (0.200)
R_free_	0.184 (0.251)
RMSD bond lengths	0.014 Å
RMSD bond angles	1.63°
Ramachandran	
Favored	100.0%

### 2.3 Structure determination, refinement, and analysis

Data were processed using HKL2000 [37] (**Table 1**) and the structure determined in the Phaser subroutine [38, 39] of PHENIX [40] using the SH3 domain of Lck as the search model [41]. Model improvement used alternating rounds of model building in Coot [42, 43] and refinement in PHENIX [40]. Two distinct positions of the C-terminus (N122 to H126) were observed in the electron density, which likely resulted in higher R-values (**Table 1**). The model of the C-terminus focused on the conformation with better electron density but could not be modeled with accuracy, and contains two rotameric outliers. Structural superpositions were performed in Coot [42, 43] and figures were rendered in PyMOL Molecular Graphics System, Version 2.0 Schrödinger, LLC (New York, NY).

## Results and discussion

### Structure of the Lyn SH3 domain

Like all Src-family kinases, the multi-domain architecture of Lyn makes the determination of a structure of the full-length protein a significant challenge because the individual domains are predicted to move relative to each other upon kinase activation [44]. Although large, multi-domain fragments of Src, Hck, Lck and Fyn from the Src-family of kinases have been reported [44–48], structural investigations of Src-family kinases still benefit from the use of isolated domains. For the Lyn kinase, prior structural work includes crystal structures of the kinase domain [49, 50] and the SH2 domain [51] as well as NMR structures of both the unliganded and liganded SH3 domain [36].

**Fig 1:**
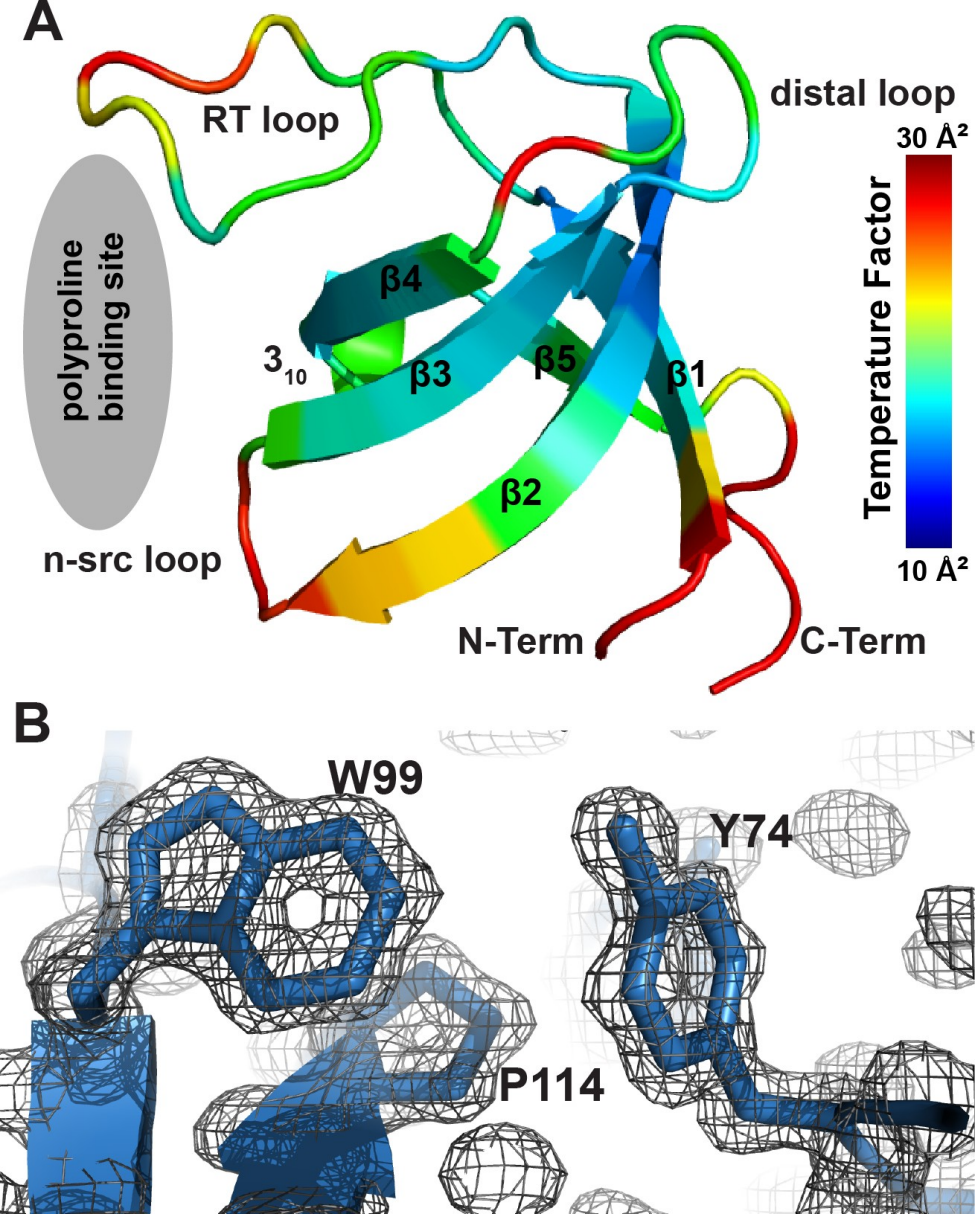
Crystal structure of the Lyn SH3 domain. **A.** Ribbons representation of the Lyn SH3 domain highlights the canonical β-barrel. The structure is colored by crystallographic temperature factor. **B.** 2*F*_*o*_*−F*_*c*_ electron density map contoured at 2.0 σ and rendered around Y74, W99 and P114. These residues contribute to the binding site for the polyproline motif [36] and are critical for protein-protein interactions.

**Fig 2:**
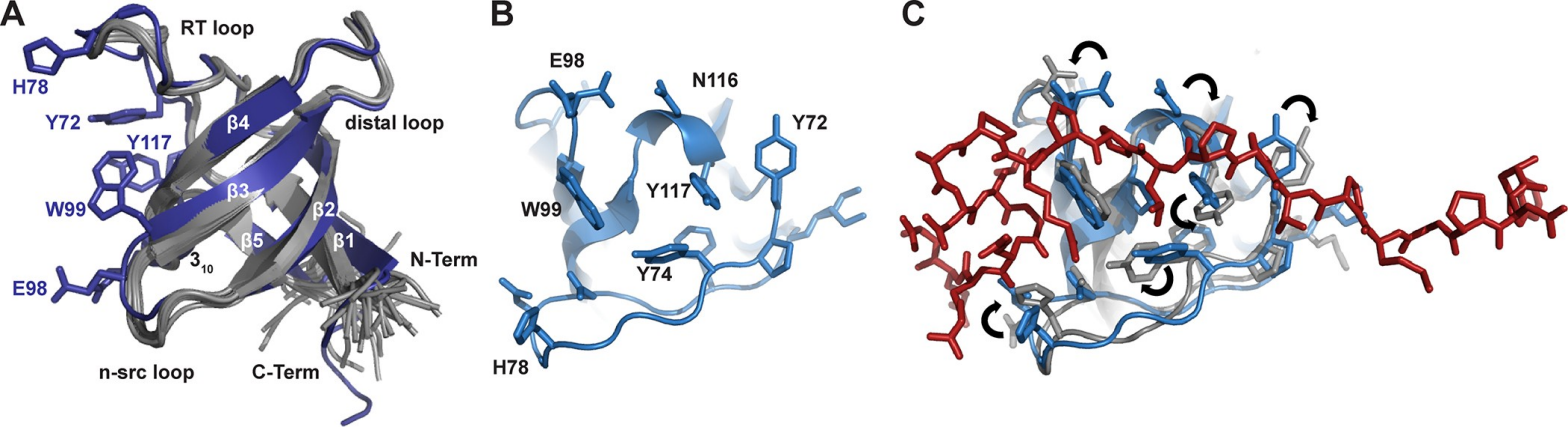
Comparison of the Lyn SH3 structures. **A.** Overlay of the X-ray structure with the NMR structure. The Lyn SH3 crystal structure is colored in blue and the NMR structures in grey. The RMSD value is 0.93 Å for all Cα atoms. **B.** Comparison of the crystal structure of the unliganded Lyn SH3 domain with the NMR structure with peptide bound. Top view of the polyproline binding pocket of the X-ray crystal structure of the Lyn SH3 domain. The highlighted residues are in different conformations than observed in the peptide-bound NMR structure. **C.** Overlay of the unliganded X-ray crystal structure of unliganded SH3 domain (blue) with peptide-bound NMR structure (grey). The peptide is shown in red. Side chain rotations are indicated by arrows.

The crystal structure of the Lyn SH3 domain (**Fig 1A and 1B**) contains five β-strands organized into two β-sheets. Previously termed β1 –β5, these β-strands are connected via four loops termed the RT loop, n-src loop, distal loop, and 3_10_ helical loop. Comparison of our crystal structure with the structure of the unliganded Lyn SH3 domain determined by NMR spectroscopy (PDB 1WIF) [36] yields an RMSD of the Cα atoms of 0.76 Å for residues of the β-barrel and 0.93 Å for all Cα atoms (**Fig 2A**). The largest differences in position involve the residues that are found within the polyproline binding cleft, G76, H78 and P79 of the RT loop and E98, E99 of n-src loop, which exhibit positional differences of 1.5 Å – 2.3 Å due to rotation of these loop elements. Conformational changes in these two loops are observed in the NMR structure bound to the herpesvirus-derived peptide [36] (**Fig 2B and 2C**).

Flexibility of the RT and n-src loops has previously been suggested as important for interaction with polyproline motifs, which might proceed via an induced fit mechanism [52–55]. These movements are expected to optimize the contacts between the SH3 domain and the polyproline motif. This could allow for variations on the polyproline sequence motifs that can selectively interact with particular SH3 domains. In the case of the Lyn kinase, physiological partners include PI-3 kinase and Ras-GAP [5, 6]. These binding partners are proposed to alter the position of the SH3 domain in the full-length Src-family kinases, which increases kinase activity.

### Cancer-associated mutations

In the absence of a mutation or a protein-protein interaction, the SH3 domain of Src family kinases binds to the kinase domain and inhibits activity [45, 56]. Physiologically, protein-protein interactions with the SH3 domain are believed to move its position so that it no longer inhibits the activity [56, 57]. Thus, the cancer-associated mutations in the Lyn SH3 domain could increase Lyn activity by eliminating this self-inhibition.

**Fig 3:**
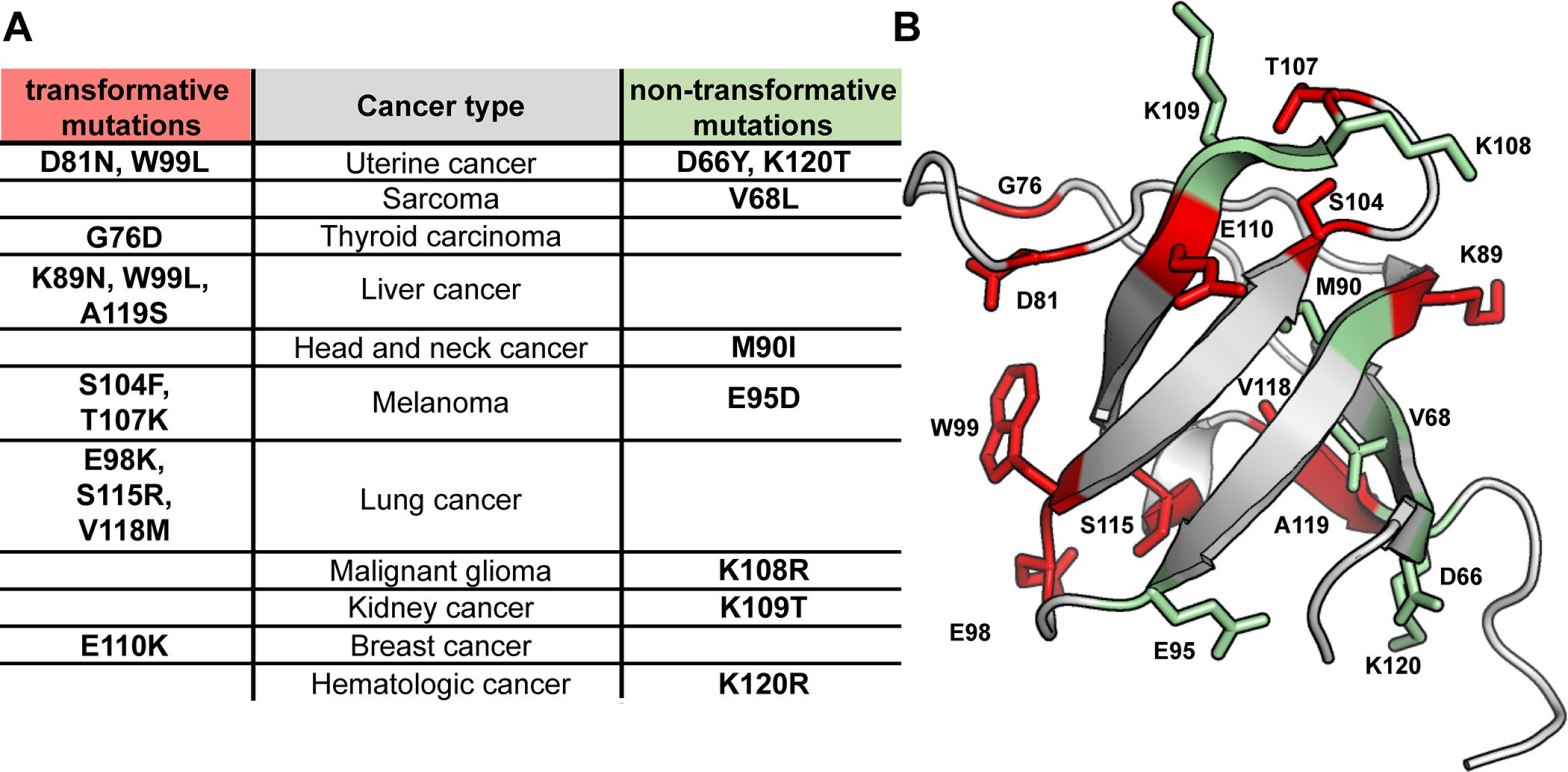
Cancer-associated mutations of the Lyn SH3 domain. **A.** Mutations of 18 out of the 61 amino acids of the Lyn SH3 domain were identified by genome sequencing of patients with the indicated types of cancer [23–25]. The table separates these mutations into likely-transformative and likely non-transformative, based upon prior computational analysis [58]. **B.** Locations of the likely transformative cancer-associated mutations are shown in *red*. Locations of the likely non-transformative substitutions are shown in *green*.

Advances in sequencing now allow mutations in individual cancer patients to be identified. Analysis of whole genomes revealed 18 cancer-associated point mutations within the Lyn SH3 domain, affecting ~30% of the residues in this domain [23–25] (**Fig 3**). While cell assays evaluating the impact of these single amino acid changes have not, to date, been reported, the use of computational prediction algorithms suggests that 12 of these substitutions are likely to be transformative and contribute to disease.

Evaluation of the locations of these mutations in the context of the structure suggests that many of the likely transformative substitutions could prevent the interaction of the SH3 domain with the kinase domain, but by distinct mechanisms (**Fig 3**). For example, a subset of the mutations, including D81N, W99L and E98K, may disrupt the protein-protein interaction interface with the kinase domain, and eliminate negative regulation of kinase activity. Other mutations, such as V118M, may destabilize the fold of the SH3 domain, again preventing the normal attenuation of kinase activity in the basal state of Lyn. In contrast, single amino acid substitutions that are predicted to be non-transformative are either relatively conservative, or are located on the surface of the SH3 domain that is not engaged by the kinase domain.

## Conclusions

The crystal structure of the Lyn kinase SH3 domain improves the molecular understanding of the regulatory mechanism of the Src family kinases. The mapped cancer-associated mutations in the SH3 domain identify how different regions of this domain affect protein function.
